# Cyanogen Metabolism in Cassava Roots: Impact on Protein Synthesis and Root Development

**DOI:** 10.3389/fpls.2017.00220

**Published:** 2017-02-24

**Authors:** Tawanda Zidenga, Dimuth Siritunga, Richard T. Sayre

**Affiliations:** ^1^Bioscience Division, Los Alamos National Laboratory, Los AlamosNM, USA; ^2^Department of Biology, University of Puerto Rico, MayaguezPR, USA; ^3^New Mexico Consortium, Los AlamosNM, USA

**Keywords:** cassava, linamarin, cyanide, cyanogen, β-cyanoalanine synthase, nitrilase, auxin, ethylene

## Abstract

Cassava (*Manihot esculenta* Crantz), a staple crop for millions of sub-Saharan Africans, contains high levels of cyanogenic glycosides which protect it against herbivory. However, cyanogens have also been proposed to play a role in nitrogen transport from leaves to roots. Consistent with this hypothesis, analyses of the distribution and activities of enzymes involved in cyanide metabolism provides evidence for cyanide assimilation, derived from linamarin, into amino acids in cassava roots. Both β-cyanoalanine synthase (CAS) and nitrilase (NIT), two enzymes involved in cyanide assimilation to produce asparagine, were observed to have higher activities in roots compared to leaves, consistent with their proposed role in reduced nitrogen assimilation. In addition, rhodanese activity was not detected in cassava roots, indicating that this competing means for cyanide metabolism was not a factor in cyanide detoxification. In contrast, leaves had sufficient rhodanese activity to compete with cyanide assimilation into amino acids. Using transgenic low cyanogen plants, it was shown that reducing root cyanogen levels is associated with elevated root nitrate reductase activity, presumably to compensate for the loss of reduced nitrogen from cyanogens. Finally, we overexpressed *Arabidopsis CAS* and *NIT4* genes in cassava roots to study the feasibility of enhancing root cyanide assimilation into protein. Optimal overexpression of *CAS* and *NIT4* resulted in up to a 50% increase in root total amino acids and a 9% increase in root protein accumulation. However, plant growth and morphology was altered in plants overexpressing these enzymes, demonstrating a complex interaction between cyanide metabolism and hormonal regulation of plant growth.

## Introduction

Cyanide is ubiquitous in nature and as such most eukaryotic organisms have developed mechanisms for its detoxification. For example, all plants produce some level of cyanide as a byproduct of the ethylene biosynthesis (Peiser et al., 1984; Dong et al., 1992). Additionally, some plants are highly cyanogenic including; cassava, bitter almonds, and rubber (Poulton, 1990; Gleadow and Møller, 2014). The leaves and roots of cassava plants may accumulate between 200 and 1,300 mg CN equivalents/kg dry weight (Siritunga and Sayre, 2004). Generation of free cyanide from cyanogenic compounds would obviously have toxic consequences, but generally does not happen in intact plants due to the physical separation of the cyanogenic compounds from the enzymes that degrade them (Kojima et al., 1979; Poulton, 1990; McMahon et al., 1995; Maruyama et al., 2001). In cassava, the cyanogenic glycoside linamarin, is stored in vacuoles while its corresponding β-glucosidase, linamarase, is localized to the cell wall and laticifers (Mkpong et al., 1990; McMahon et al., 1995; Elias et al., 1997a). Tissue disruption, e.g., during mechanical damage, initiates hydrolysis of linamarin by the generalized β-glucosidase, linamarase, to produce acetone cyanohydrin. Acetone cyanohydrin can spontaneously decompose to yield cyanide and acetone at pH > 5.0 or temperatures > 35°C, or is broken down by the enzyme hydroxynitrile lyase (HNL), which is expressed only in cassava leaves and stems and not in roots (White et al., 1998).

The cassava tuberous root, the main consumed part of the plant, is actually a true root, not a tuber, and thus cannot be used to vegetatively propagate the plant (Alves, 2002). Chronic, low-level dietary cyanide exposure associated with the consumption of improperly processed cassava can lead to several health disorders, including tropical ataxic neuropathy, characterized by optic atrophy and an inability to coordinate muscle movements, and a paralytic disorder known as Konzo (Osuntokun, 1981; Rosling, 1994; Tylleskar, 1994; Adamolekun, 2010). Individuals with these disorders typically have very low concentrations of sulfur amino acids in the blood (available sulfur is preferentially used in cyanide detoxification by rhodanese) and elevated levels of plasma thiocyanate (Shibamoto and Bjeldanes, 1993; Adamolekun, 2010).

For obvious health impact reasons, several efforts have been directed toward lowering the cyanogens (a group of nitrile-containing plant secondary compounds that release hydrogen cyanide through enzymatic activity) in cassava. The major cyanogens in cassava are linamarin and acetone cyanohydrin. Transgenic cassava lines having less than 1% of normal root cyanogen levels have been generated by inhibiting linamarin biosynthesis in leaves (Siritunga and Sayre, 2003). However, these plants could not grow without ammonia and generally produced smaller tuberous roots compared to wild-type plants. It was hypothesized that the poor performance of these plants was due to the suppression of cyanogen synthesis, an important source of reduced nitrogen for roots. A second approach to reduce cyanogen levels in cassava foods was to overexpress HNL to accelerate the conversion of acetone cyanohydrin into cyanide, which is then volatilized during processing (Siritunga et al., 2004; Narayanan et al., 2011). Both acetone cyanohydrin and linamarin contribute to cyanide toxicity in poorly processed cassava foods, whereas cyanide does not due to its volatilization. Significantly, by increasing root nitrogen sink strength by overexpressing HNL, it was observed that there was a 50–75% reduction in root steady-state linamarin levels suggesting that linamarin provided reduced nitrogen for protein synthesis.

**FIGURE 1:**
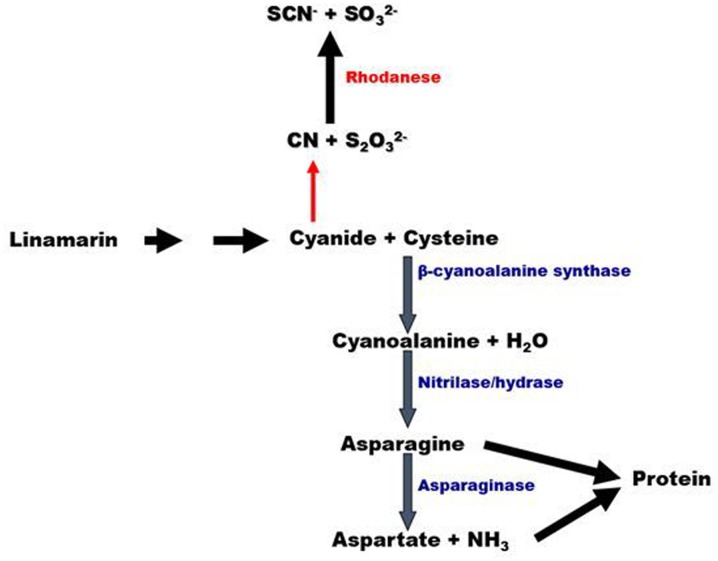
**Model of cyanide assimilation in plants.** CAS converts cyanide released from the cyanogen linamarin, to cyanoalanine plus hydrogen sulfide in the presence of cysteine. Cyanoalanine is converted by a nitrilase to asparagine, which is then converted to aspartate and ammonia by asparaginase. In this way, cyanide nitrogen can be incorporated into the free amino acid pool of the plant. An alternative pathway (red arrow) can potentially result in cyanide detoxified to thiocyanate by rhodanese.

It had been proposed that some fraction of the cyanogens transported from leave to roots were metabolized to generate free cyanide which would then either be detoxified by the enzyme rhodanese (cyanide: thiosulfate sulfurtransferase) or assimilated into amino acids by CAS and nitrilase (Siritunga and Sayre, 2007; **Figure 1**). CAS catalyzes the reaction between cyanide and cysteine to form β-cyanoalanine and hydrogen sulfide. This detoxification pathway results in the assimilation of cyanide into amino acid biosynthesis pathways and is present in all higher plants thus far examined (**Figure 1**; Blumenthal et al., 1968; Miller and Conn, 1980).

In this study, we use a combination of biochemical assays and transgene expression studies to demonstrate that cyanide metabolism in roots most likely occurs via the CAS pathway to produce amino acids. Furthermore, we show no competing rhodanese activity in roots, consistent with CAS mediated assimilation of cyanide into amino acids. Furthermore, overexpression of CAS and nitrilase was shown to lead to elevated free amino acid pool sizes and root protein content under optimal enzyme expression levels, indicating that these enzymes facilitate cyanide assimilation. These results provide insights into the role for cyanogenic glycosides in nitrogen metabolism in the roots, as well as possibilities for redirecting root linamarin toward protein production. Finally, alterations in cyanide assimilation and other metabolic pathways associated with overexpressing CAS and NIT4 was shown to dramatically alter plant growth and morphology. These results demonstrate a potential complex interaction between cyanide and ethylene and auxin metabolism.

## Materials and Methods

### Cassava Cultivars

Cassava cultivar *Manihot Columbia* 2215 (MCol 2215) was used for initial assays of leaf and root enzyme activity. Transformation work was done using cultivar TMS 60444, selected due to ease of transformation. Comparative enzyme assays with transgenic lines were performed using TMS 60444 as the wild-type control. Transgenic lines previously generated for low cyanogenesis (by selectively inhibiting cyanogenic glycoside synthesis in the leaves), Cab1-1, Cab1-2, and Cab1-3 used MCol 2215 as the background (Siritunga and Sayre, 2003).

### Tissue Culture Propagation of Plant Material

Cassava plants were propagated *in vitro* on the Murashige and Skoog (MS) basal medium (Murashige and Skoog, 1962) supplemented with Gamborg vitamins (Gamborg et al., 1968) and 2% (w/v) sucrose. *In vitro* plants were propagated in growth incubators at 28°C with a photoperiod of 16 h of light and 8 h of darkness. Micropropagation of plant materials was done once every 5–8 weeks depending on the requirements of specific experiments (Siritunga and Sayre, 2003; Ihemere et al., 2006; Zidenga et al., 2012).

### Total Protein Extraction and Analysis

Total protein was measured using the Bradford assay according to the supplier’s (Invitrogen^1^) instructions. Protein was extracted from root and leaf tissue of cassava using 50 mM Tris-HCl (pH 8.5), 5 mM dithiothreitol and 1 mM EDTA. Extraction buffer was used at a ratio of 5 mL of buffer per gram fresh tissue. Leaves were ground in liquid nitrogen to a fine powder before adding the buffer. Tuberous roots were blended together in the buffer in the Magic Bullet MB1001 blender (Homeland Houseware LLC) for 30 s at 4°C. The ground extract was passed through four layers of cheesecloth and centrifuged at 21000 *g* for 10 min. The supernatant was used as the crude extract and measured for protein using the Bradford reagent with bovine serum albumin (BSA) as the standard.

### Activity of β-Cyanoalanine Synthase (CAS) in Cassava Tissue

β-Cyanoalanine synthase activity was determined using the method described by Goudey et al. (1989) with some modifications. Cassava tissue was ground in liquid nitrogen using a motor and pestle and extracted in a buffer containing 50 mM Tris-HCl, 5.0 mM dithiothreitol (DTT), 5.0 mM phenylmethylsulfonyl fluoride (PMSF) and 1.0 mM EDTA at pH 8.0. The extract was filtered through four layers of cheesecloth to remove debris. To 500 μL of substrate solution (10 mM L-cysteine and 10 mM NaCN in 50 mM Tris buffer pH 8.0), and 100–200 μg of crude protein extract was added to make a total reaction volume of 1.0 mL. The optimum concentration of enzyme used was determined after testing different amounts of wild-type plant enzyme extract. The reaction was carried out for 10 min and stopped by adding 0.1 mL of 30 mM ferric chloride in 1.2 N HCl followed by 0.1 mL of 20 mM *N,N* dimethyl-*p*-phenylenediamine sulfate in 7.2 N HCl. Absorbance was measured at 640 nm after 10 min. Concentration of sodium sulfide (Na_2_S) was estimated from the absorbance using a standard curve prepared by adding 0.1 mL 30 mM ferric chloride in 1.2 N HCl per mL of total reaction mixture followed by 0.1 mL of 20 mM *N,N* dimethyl-*p*-phenylenediamine sulfate in 7.2 N HCl to 1 mL of known concentrations of Na_2_S.

### Rhodanese Activity

Rhodanese activity was assayed as described by Wang and Volini (1968) with modifications. Cassava plant tissue was ground in liquid nitrogen using a motor and pestle and extracted in 200 mM sodium phosphate buffer pH 7.8, 5.0 mM DTT, 5.0 mM PMSF, 1.0 mM EDTA, 1.0 mM Na thiosulfate (to keep the enzyme in a stable rhodanese-sulfur intermediate), 5 mM KCl and 2% w/v polyvynilpolypyrrolidone (PVP). The homogenate was passed through four layers of cheesecloth to remove debris and centrifuged at 21000 *g* for 5 min at 4°C. The supernatant was used in subsequent assays. To start the reaction, about 100–200 μg of protein was added to 0.5 mL of 50 mM NaCN and 50 mM of Na thiosulfate in 200 mM sodium phosphate buffer (pH 7.8) to a total volume of 1.0 mL. The reaction was incubated at 30°C for 10 min and stopped by adding 0.5 mL 15% (v/v) formaldehyde. Absorbance at 460 nm was measured after adding 2.5 ml of ferric nitrate reagent. The reagent was prepared by adding 20 mL nitric acid (65%) to 60 mL of water, dissolving 10 g ferric nitrate: 9 H_2_O and making up to a final volume of 100 mL. The reaction was blanked using boiled (inactive) enzyme extract. The standard curve used to estimate the concentration of thiocyanate was prepared using a range of known concentrations of thiocyanate in the same volume as the reaction.

### Determination of Nitrilase Activity

Nitrilase activity was determined as described by Piotrowski et al. (2001) with some modifications. Cassava tissue was homogenized in an extraction buffer containing 50 mM Tris-HCl (pH 8.5), 2.0 mM EDTA, 8.0 mM cysteine, 2% (w/v) PVP plus and minus (for plant protein quantification) 0.1% (w/v) BSA. Tuberous greenhouse roots were homogenized in a blender for 5 s × 2 s, while *in vitro* plant material was homogenized by grinding with liquid nitrogen in a motor and pestle. In all cases, the homogenate was filtered through four layers of cheesecloth and centrifuged for 5 min at 22000 *g*. Approximately 400 μg of plant protein was used in the subsequent enzyme assay. Enzyme extracts were pre-warmed at 37°C for 2 min before being incubated with substrate (10 mM cyanoalanine in 50 mM Tris-HCl, pH 8.5 and 1.0 mM DTT) for 10 min at 37°C. The total reaction volume was 1.0 mL. The reaction was stopped by adding 100 μL of tricarboxylic acid and centrifuged at 22000 *g* for 2 min. To 500 μL of the supernatant, 1.0 mL of Nessler’s reagent (Sigma-Aldrich^2^) was added. The samples were incubated at room temperature for 10 min to allow color development. For blank samples, TCA was added at time 0. Absorbance was read at 480 nm and the amount of ammonia produced was estimated using a standard curve.

### Nitrate Reductase Activity

Nitrate reductase was assayed using the method described by the Nitrate Elimination, Co., Inc. (NECi^3^) with some modifications. Crude protein was extracted from the ground tissue using an extraction buffer containing 100 mM 3-(*N*-morpholino)propanesulfonic acid (MOPS) pH 7.5, 1.0 mM EDTA and 10 mM L-cysteine. PVPP [1% (w/v)] was added to the grinding mixture during extraction. Four mL of extraction buffer was used per gram fresh weight plant tissue. The homogenate was passed through four layers of cheesecloth and centrifuged at 21000 *g* for 5 min at 4°C. The supernatant was used in subsequent assays. Approximately 100–200 μg of the extracted protein was added to 800 μL of substrate solution (30 mM potassium nitrate in 100 mM MOPS, pH 7.5). The reaction was started by adding 100 μL of 25 mM NADH and stopped after 10 min by adding 100 μL of 100 mM zinc acetate. After centrifuging at 22000 *g* for 2 min, 500 μL of the supernatant was add to a fresh 1.5 mL tube. To this, 500 μL (an equal volume to the volume of supernatant) was added of each of the color development reagents [1% (w/v) sulfanilamide in 1.5 N HCl and 0.02% *N*-(napththyl)-ethylenediaminehydrochloride]. Samples were left at room temperature for 10–20 min to allow full color development. Absorbance was read at 540 nm. Nitrite concentration was estimated using a standard curve prepared by diluting known concentrations of nitrite in 500 μL and adding the color development reagents.

### Transformation of Cassava with the β-Cyanoalanine Synthase and Nitrilase Genes

#### Construct Design

Constructs were assembled as previously described in Zidenga et al. (2012). A modified pBI121 plasmid with a 1.2 kb *Solanum tuberosum* class I patatin promoter was used for both constructs (Ihemere, 2002; Siritunga and Sayre, 2003). The *CAS* and *NIT4* (TAIR: At5g22300) genes of *Arabidopsis* were received from the *Arabidopsis* Biological Research Center^4^ in the pUNI51 vector and cloned into SmaI and SstI restriction sites of the modified pBI121 binary plasmid (**Figure 3A**).

#### Cassava Transformation

Somatic embryogenesis, co-cultivation with *Agrobacterium* and plant regeneration were carried out as described by Zidenga et al. (2012) while cassava transformation was done following the method described by Taylor et al. (1996) with modifications (Zidenga et al., 2012).

#### RT-PCR Analysis of Transgenic Plants

RNA was isolated from 100 mg of cassava roots using the Qiagen RNeasy Plant Mini kit (Qiagen, Inc., Valencia, CA, USA). To quantify RNA, absorbance was measured at 260 and 280 nm (Sambrook et al., 1989). Concentrations of RNA were calculated based on absorbance at 260 nm. RNA purity was judged based on the 260/280 ratio where pure RNA has a value of 2. Prior to cDNA synthesis, the RNA was treated to remove DNA contamination using the Promega DNAse treatment (Promega Corporation, Madison, WI, USA). About 2–10 μg of RNA was used for cDNA synthesis using the Qscript cDNA kit (Quanta Biosciences, Gaithersburg, MD, USA).

The cDNA was used to check for the expression of the transgene by RT-PCR. For *CAS*, the forward primer was CATGCTATCACAGGCAATGG while the reverse primer was GCCAAATGTTTG AACGATCGG. For *NIT4* the forward primer was GCACTTGAGGGTGGATGTTT and the reverse was GCCAAATGTTTG AACGATCGG. For tubulin control, the primers *TubF* (TATATGGCC AAGTGCGATCCTCGACA) and *TubR* (TTACTCTTCATAATCCTTCTCAAGGG) were used as positive controls for the PCR reaction. The PCR reaction conditions were based on Choice^TM^ Taq DNA polymerase from Denville Scientific, Inc.^5^

### Plant Growth in the Greenhouse

Greenhouse plants were grown as described by Zidenga et al. (2012).

### Measurement of Yield Parameters

Plants were grown in the greenhouse in rectangular trays with only six plants per tray. Greenhouse grown plants were harvested after 4–8 months of growth and fresh weight measurements were taken on all the tuberous roots.

### Free and Hydrolyzed Amino Acid Extraction and Analysis

Free amino acid extraction was based on the method by Hacham et al. (2002). Approximately 150 mg of tissue was ground in liquid nitrogen and homogenized by motor pestle with 600 μL of water: chloroform: methanol (3:5:12 v/v). After centrifugation at 21000 *g* for 2 min, the supernatant was collected and the residue was re-extracted with 600 μL of water: chloroform: methanol followed by centrifugation. Supernatants from the first and second extraction were combined in a 2 mL tube. 300 μL of chloroform and 450 μL of water were added followed by centrifugation at 21000 *g* for 2 min. The upper water: methanol phase was collected and transferred to a fresh tube, dried by speed-vac and dissolved in 200 μL of water. Detection of free amino acids was performed by the Proteomics and Mass Spectrometry Facility at the Donald Danforth Plant Science Center using the AccQTag system. Protein hydrolysis was carried out as described by Narayanan et al. (2011). Samples were hydrolyzed for 24 h at 116°C in 6 N HCl containing 0.5% (v/v) phenol, dried and resuspended in 20 mM HCl before derivatization with the AccQ-tag reagent and subsequent separation by ACQUITY UPLC^®^ System (Waters, Milford, MA, USA) according to manufacturer’s instructions.

### Analysis of IAA

Indole acetic acid (IAA) analysis was carried out using an LC–MS/MS analysis method developed and performed by the Proteomics and Mass Spectrometry Facility at the Donald Danforth Plant Science Center. The method is similar to Chen et al. (2009), but modified to include additional plant hormone species.

### Statistical Analysis

Statistical analysis was carried out using GraphPad Prism software package^6^. Student’s *t*-test and one-way ANOVA with Dunnett’s Multiple Comparison test for comparing multiple lines with the control were used. All analyses for significant differences were performed at *P* ≤ 0.05.

## Results

### Cyanide Metabolism in Cassava Roots Occurs via β-Cyanoalanine Synthase

Cyanide is detoxified in plants either through condensation with cysteine, catalyzed by CAS, or via condensation with thiosulfate, derived from sulfur metabolism, and catalyzed by the enzyme rhodanese (**Figure 1**; Hatzfeld and Saito, 2000). Cyanide detoxification by rhodanese is prominent in mammals where the thiocyanate is excreted in urine (Shibamoto and Bjeldanes, 1993). The use of thiosulfate as a cyanide antidote is based on the rhodanese activity (Shibamoto and Bjeldanes, 1993; Shepherd and Velez, 2008). In plants, however, the relationship between rhodanese and cyanide metabolism has not been firmly established (Chew, 1973; Miller and Conn, 1980; Hatzfeld and Saito, 2000). Relevant to this observation it has been demonstrated that rhodanese activity does not correlate with cyanogenic potential (Miller and Conn, 1980). It has been suggested, however, that thiocyanate, derived from rhodanese activity could be hydrolyzed by a thiocyanate hydrolase to generate ammonia and carbonyl sulfide in plants, but this activity has yet to be confirmed (Yu et al., 2012).

**FIGURE 2:**
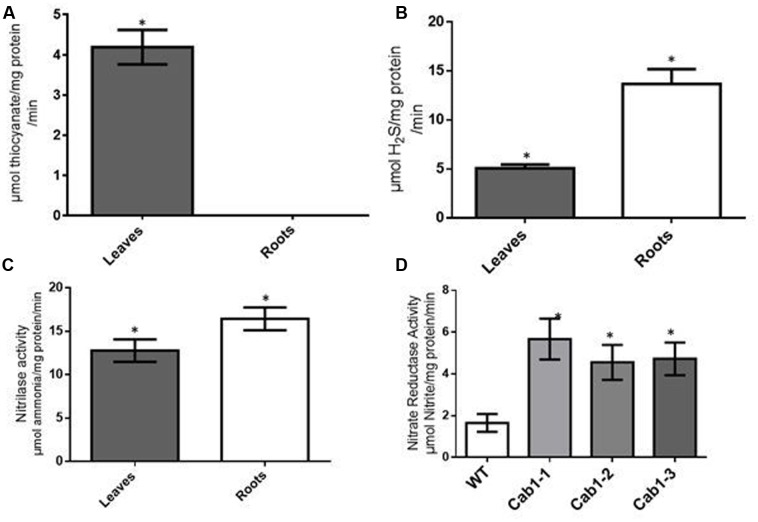
**Cyanide metabolizing enzymes (except rhodanese) in cassava have higher activities in roots compared to leaves.** **(A)** Activities of rhodanese (in μmol thiocyanate/mg protein/minute) in tuberous roots and leaves of 8 months old cassava plants grown under glasshouse conditions. **(B)** CAS activity (in μmol hydrogen sulfide (H_2_S) per mg protein per minute) in 8 months old cassava **(C)**. Nitrilase (cyanoalanine hydratase) enzyme activities (in μmol ammonia/mg protein/min) in roots and leaves of *in vitro* cassava plants at 5 weeks. **(D)** Analysis of nitrate reductase activity (μmol nitrite/mg protein/min) in wild-type (WT) and transgenic low cyanogen (Cab1-1, Cab1-2, and Cab1-3; Siritunga and Sayre, 2003) lines. The assay was conducted on 5 weeks old *in vitro* plants. The results of all experiments are the averages from biological four trials. Statistical analysis was done by one-way ANOVA with Dunnett’s Multiple Comparison Test. Asterisks indicate significant difference at *P* ≤ 0.05.

To determine whether rhodanese plays a role in cyanide detoxification in cassava, we measured rhodanese activity in leaves and tuberous roots of the cassava. The average rhodanese activity detected in cassava leaves was relatively low at 4.19 μmol/mg protein/min (**Figure 2A**). Significantly, we detected no rhodanese activity in cassava tuberous roots (**Figure 2A**). Since cyanogen accumulation occurs in roots, the lack of rhodanese activity in these tissues suggests that rhodanese is not involved in cyanide metabolism in roots. As previously discussed, linamarin is produced in the leaves and transported to the roots where it presumably provides reduced nitrogen for protein synthesis (Bediako et al., 1981; Ramanujam and Indira, 1984; Siritunga and Sayre, 2003; Siritunga et al., 2004). Since no rhodanese activity was detected in cassava roots, we hypothesized that cyanide released from linamarin breakdown is preferentially assimilated via CAS. To test this hypothesis, we analyzed CAS activity in cassava tuberous roots and leaves. If cyanogens are a source of reduced nitrogen for protein synthesis in roots, we would expect that CAS activity (unlike rhodanese activity) would be higher in cassava roots than leaves. In cassava tuberous roots, average CAS activity was 13.7 μmol HS/mg protein/min, compared to 5.1 in leaves, an approximately threefold higher CAS activity in roots than leaves (**Figure 2B**). In contrast, in potato, a non-cyanogenic plant, leaf CAS activity (≈0.04 μmol H_2_S per mg protein per min) was substantially lower than in cassava and twofold greater than potato tuber activity (≈0.02 μmol H_2_S per mg protein per min). These rates in potato correlate well with levels required for CN detoxification associated with ethylene biosynthesis (Maruyama et al., 2001). Similar differences (3X) in CAS activity between cassava roots and leaves had previously been reported by Nambisan and Sundaresan (1994) and Elias et al. (1997b).

If cyanide assimilation occurs through the CAS, then it would be expected that additional enzymes in the cyanogen assimilation pathway would have enzymatic activities commensurate with CAS activity. Thus, we assayed nitrilase activity, the enzyme involved in the conversion of cyanoalanine, the product of CAS activity, into aspartate, asparagine, and ammonia. The average nitrilase activity in cassava leaves was 12.8 μmol ammonia/mg protein per min while that in the roots was 16.4 μmol ammonia/mg protein per min (**Figure 2C**). These apparent nitrilase activity levels were similar to those observed for CAS in cassava roots. In addition, root nitrilase activity was about 1.3 times (or 30%) greater than in leaves. These observations are consistent with root cyanide assimilation by CAS and nitrilase leading to amino acid synthesis (Siritunga and Sayre, 2003).

### Compensation for Reduced Cyanogens in Low Cyanide Transgenic Plants and the Impact of Elevating Cyanide Assimilatory Enzymes on Amino Acid and Protein Levels

Previously, transgenic plants having low root cyanogen levels were generated by suppressing leaf linamarin biosynthesis through antisense expression of CYP79D1/D2 genes encoding two cytochrome P450s that catalyze the first dedicated step in cyanogenic glycoside synthesis, resulting in a 99% reduction in root linamarin levels relative to wild-type plants (Andersen et al., 2000; Siritunga and Sayre, 2003). These results confirmed that cyanogenic glycosides were transported from leaves to roots, as demonstrated also in rubber tree (Selmar et al., 1988). We have used the transgenic low root linamarin plants as tools to study cyanide metabolism in cassava. As suggested by biochemical assays described above, cyanide assimilation via CAS allows for entry of cyanide into amino acid synthesis. To determine if disruption of linamarin metabolism impacted the activity of select enzymes involved in nitrogen metabolism, we compared root nitrate reductase activity between low (transgenic) and high cyanogen cassava plants. Nitrate reductase activity is highly regulated in plants, is up-regulated in plants with reduced nitrogen availability, and repressed in plants with sufficient ammonia (Campbell, 1999). Wild-type cassava roots were observed to have an average nitrate reductase activity of 1.78 μmol nitrite/mg protein/min while low cyanogen (Cab1, **Figure 2D**) lines had nitrate reductase rates ranging from 4.5 to 5 μmol nitrite/mg protein/minute, or three times higher than wild-type (**Figure 2D**). These data suggest that when cyanogen synthesis is reduced, other root-based nitrogen assimilation pathways compensate.

Thus, we hypothesized that enhancing cyanide assimilation via overexpressing enzymes in the CAS pathway (**Figure 1**) could result in elevated root total amino acids or protein levels.

**FIGURE 3:**
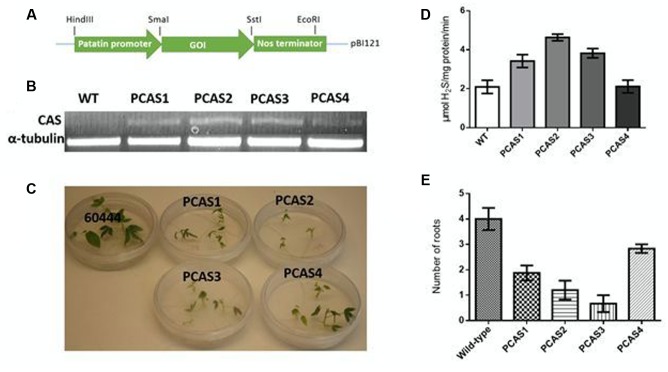
**(A)** Gene cassette used in transforming cassava with β-cyanoalanine synthase (*CAS*) and nitrilase genes. The construct was assembled in a modified pBI121 plasmid where the gene of interest (GOI) was driven by a class I patatin promoter for root-specific expression. **(B)** CAS transcripts in wild-type (WT) and transgenic (PCAS1-4) cassava lines as detected by RT-PCR. RNA was extracted from 100 mg of 5 week-old *in vitro* cassava roots. RT-PCR was performed using primers for the *CAS* insert with *tubulin* as the internal control. **(C)** *In vitro* growth comparison of transgenic *CAS*-overexpressing plants (PCAS1-4) and wild-type (TMS 60444) plants after 3 weeks. **(D)** Expression of *CAS* increased the activity of the enzyme in cassava roots. The activity of CAS was correlated to reduced growth and root development **(C,E)**. **(E)** Root development in *in vitro* transgenic *CAS* plants grown in MS medium for 3 weeks. Data are averages of *n* = 20. Statistical analysis was done by one-way ANOVA with Dunnett’s Multiple Comparison Test. All transgenics were significantly different from wild-type at *P* ≤ 0.05.

Four transgenic lines were generated overexpressing *CAS* as confirmed by RT-PCR (**Figures 3A,B**). To determine if *CAS* overexpression resulted in increased enzyme activity, CAS enzyme activity was assessed. Root CAS activity in transgenic plants was elevated as much as twofold relative to wild-type roots (**Figure 3D**).

Unexpectedly, however, we observed reduced recovery of transgenic plants overexpressing *CAS* relative to transgenics expressing other genes of interest in cassava roots (e.g., Siritunga and Sayre, 2003; Ihemere et al., 2006; Zidenga et al., 2012), suggesting negative effects of *CAS* overexpression. Of the recovered *CAS* transgenic lines, the lines with higher CAS activity showed more stunted growth (**Figure 3C**). Analysis of transgenic PCAS plants indicated poor root development compared to wild-type plants (**Figure 3E**), especially during the first 4 weeks of growth. Wild-type plants had an average of 4 roots per plant, while PCAS transgenics ranged from 1 to 3 roots per plant. Root development was poorest in PCAS2 and PCAS3. Poor root development was also associated with reduced fresh weight and poor growth. There was a 2 to 4-fold decrease in fresh weight in transgenic PCAS plants (with the exception of PCAS4, the line with the enzyme activity closest to wild-type) relative to wild-type plants. The transgenic plants exhibiting the highest CAS activity, had the lowest fresh weight. These data suggest that overexpression of *CAS* impairs growth and development in cassava plants.

To determine if *CAS* overexpression impacted amino acid pool sizes in cassava roots, total free amino acids and those obtained from hydrolyzed proteins were assessed. There was a significant difference (at *P* ≤ 0.05) in total amino acids between wild-type and transgenic plants (**Table 1**). In addition, total root protein content was increased up to 9.3% in *CAS* transgenic plants relative to wild-type (**Table 2**) as were total free and protein amino acids including most notably arginine, aspartate, and glutamate in the PCAS1 transformant, which had the lowest increases in CAS activity (**Table 1**). Since aspartate, glutamate, and arginine are entry points for reduced nitrogen assimilation and transfer, respectively, into amino acids it is not unexpected that their levels would be increased by enhancing cyanide assimilation into amino acids by CAS.

**Table 1:** Hydrolyzed and free amino acid content of 4 months-old transgenic and wild-type cassava tuberous roots in pmole/mg dry weight.

Amino acid	WT	PCAS1	PCAS2
CyA	1.30 ± 0.05	1.49 ± 0.19	1.40 ± 0.08
His	0.56 ± 0.17	0.7 ± 0.22	0.7 ± 0.2
Ser	1.63 ± 0.4	2.08 ± 0.42	2.19 ± 0.28^a^
Arg	1.30 ± 0.21	2.68 ± 0.54^a^	1.83 ± 0.25^ab^
Gly	4.01 ± 0.18	4.87 ± 1.01	5.56 ± 0.45^a^
Asp	6.56 ± 0.78	9.12 ± 0.92^a^	8.43 ± 0.86^a^
MetS	1.64 ± 0.25	1.74 ± 0.3	1.86 ± 0.11
Glu	7.27 ± 0.35	9.76 ± 1.07^a^	9.92 ± 0.88^a^
Thr	1.62 ± 0.32	1.98 ± 0.51	2.25 ± 0.15^a^
Ala	3.93 ± 0.28	4.49 ± 1	4.96 ± 0.34^a^
Pro	2.05 ± 0.17	2.41 ± 0.56	2.67 ± 0.3
Lys	3.01 ± 0.16	3.62 ± 0.69	3.87 ± 0.19^a^
Val	2.78 ± 0.19	3.32 ± 0.74	3.64 ± 0.28^a^
Ile	2.01 ± 0.11	2.35 ± 0.48	2.58 ± 0.17^a^
Leu	3.0 ± 0.16	3.51 ± 0.73	3.89 ± 0.26^a^
Phe	1.62 ± 0.09	1.78 ± 0.32	1.86 ± 0.14^a^
Total	44.3	55.9^a^	57.6^a^


*Superscript ‘a’ indicates significant difference from wild-type at *P* ≤ 0.05. Superscript ‘b’ indicates significant difference between the two transgenic lines at *P* ≤ 0.05. Values are ± standard deviation. CyA, cysteic acid (hydrolysis product of cysteine); Asp, Asp+Asn (due to hydrolysis of Asn to Asp); Glu, Glu+Gln (due to hydrolysis of Gln to Glu).*

**Table 2:** Total protein comparison in 4 months-old wild-type (WT) and transgenic cassava tuberous roots.

Plant line	WT	PCAS1	PCAS2
Total protein (mg/mg dry weight)	16.73 ± 0.56^a^	17.36 ± 0.24^a^	18.28 ± 0.17^b^


*Same letter superscript indicates no significant difference at *P* ≤ 0.05. Different letter superscripts (a/b) indicate significant difference. Measurements were performed in biological triplicates. Values are ± standard deviation.*

**FIGURE 4:**
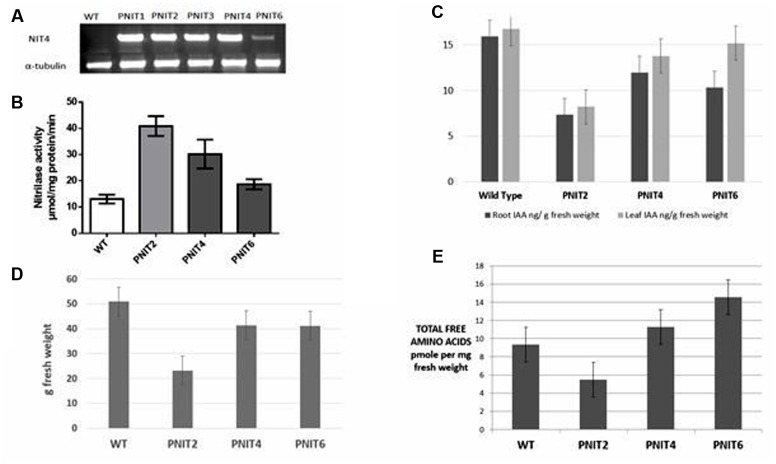
**(A)** *NIT4* transcript abundance by RT-PCR. RNA was extracted from 100 mg of 5 week-old *in vitro* cassava roots. RT-PCR was performed using primers for the *NIT4* insert while *tubulin* primers were used for the control. **(B)** Expression of Nitrilase increases cyanoalanine hydrolase activity in cassava roots. Rates of conversion of cyanoalanine to ammonia were determined for *n* = 4. **(C)** IAA analysis in 4 months-old greenhouse-grown wild-type and transgenic cassava tuberous roots and leaves showing decreased IAA in transgenic plants overexpressing *NIT4*. **(D)** Storage root fresh weight per plant in WT and transgenic *NIT4* (PNIT2, 4, and 6) lines. **(E)** Total free amino acid analysis in wild-type and *NIT4* transgenic lines. The data are averages of three biological trials.

As previously described, we observed elevated nitrilase activity in cassava roots relative to leaves. Thus, we hypothesized that NIT overexpression would increase assimilation of cyanide into amino acids (**Figure 1**). Transformation of cassava plants with the *Arabidopsis NIT4* gene was carried out as described in Section “Materials and Methods.” Transgenic lines were confirmed by RT-PCR (**Figure 4A**). To determine whether overexpression of *NIT4* in cassava roots increased nitrilase activity, enzyme assays were carried out as previously described. Three transgenic lines were used for this analysis. Total nitrilase activity in transgenic lines was increased as much as fourfold relative to wild-type plants (**Figure 4B**). Interestingly, free amino acid pool sizes increased as much as 50% in plants with NIT activities less than 3X wild-type levels but dropped to as much as 50% of wild-type levels in plants having fourfold increases in NIT activity (**Figure 4E**). These results suggest that NIT4 may have pleiotropic effects with super-elevated NIT4 activity having negative impacts on plant metabolism. To assess whether there were additional phenotypic effects of *NIT4* overexpression, we assessed their performance in the greenhouse. Interestingly, PNIT plants displayed an increased branching phenotype compared to wild-type plants (data not shown). In addition, in the early stages of growth (up to 8 weeks) greenhouse-grown PNIT plants tended to have more fibrous root development compared to wild-type plants. These phenotypic traits mimicked potential morphological responses associated with alterations in ethylene or auxin levels. It is known that auxin promotes cell division in root pericycle cells, which leads to lateral root formation, but inhibits cell division in lateral meristems of the shoot, resulting in the inhibition of lateral bud growth, or apical dominance (Rogg et al., 2001). The branching phenotype in our transgenic plants is similar to that observed upon decapitation of apical meristems which removes the inhibition of lateral bud growth (apical dominance) resulting in increased branching. Decapitation results in reduced IAA levels, the most common bioactive form of auxin (Ferguson and Beveridge, 2009). However, while a significant amount of root auxin is derived from the shoot, it is now known that roots are also sites of auxin biosynthesis (Ross et al., 2006).

Since nitrilases are also known to be involved in auxin biosynthesis, we hypothesized that *NIT4* overexpression affected auxin metabolism in cassava tuberous roots leading to the increased branching phenotype. To test this hypothesis, we measured IAA concentrations in cassava tuberous roots of 4 months-old greenhouse grown wild-type and *NIT4* transgenic plants. Transgenic cassava plants expressing *NIT4* had up to 50% less root IAA compared to wild-type plants (**Figure 4C**). Wild-type roots, with ≈16 ng/g fresh weight, had twice the level of free IAA as the highest *NIT4* expressing transgenic plants. IAA concentrations also decreased in the leaves, with PNIT2 having 50% less than wild-type levels (**Figure 4C**). The observed reduction in IAA concentration is consistent with the increased root branching but is inconsistent with the enhanced root NIT4 activity if NIT4 is expected to impact IAA levels (O’Reilly and Turner, 2003). The role of nitrilases in auxin biosynthesis is still not clearly defined (Ljung et al., 2002; Mano and Nemoto, 2012). All four *Arabidopsis* nitrilases have been shown to convert indole-3-acetonitrile (IAN) to the plant hormone IAA, but NIT4 appears to be mainly involved in cyanide metabolism (Bartling et al., 1992, 1994; Schmidt et al., 1996; Normanly et al., 1997; Piotrowski et al., 2001; Ljung et al., 2002). In our experiments, overexpression of *NIT4*, while increasing cyanide assimilation into amino acids, was associated with reduced IAA levels. It is not immediately clear how *NIT4* overexpression in roots may have decreased IAA levels, however, we observed additional phenotypic impacts of NIT4 overexpression including reduced storage root yield in transgenic lines having the highest nitrilase activity (**Figures 4B,D**). Finally, elevated total free amino acids were only observed in *NIT4* transgenics expressing the lowest levels of increased total NIT activity relative to wild-type (**Figures 4D,E**). Plants with the highest NIT activity had the lowest total free amino acid pool sizes. Thus, the regulation of amino acid accumulation by NIT is complicated with only a narrow window of enhanced NIT activity yielding enhanced amino acid accumulation.

## Discussion

Cyanogenic plants produce sufficient levels of cyanogens to provide protection against a variety of herbivores and pathogens (Nahrstedt, 1985; Jones, 1998). It is this function of cyanogens that has received the greatest attention since it can potentially impact human health. In addition to their defensive role, cyanogenic glycosides have been proposed to function as transportable forms of reduced nitrogen in some plants including; rubber tree, cassava and sorghum (Selmar et al., 1988; Poulton, 1990; Siritunga and Sayre, 2007; Gleadow and Møller, 2014). Cyanogenic plants can allocate a substantial amount of nitrogen to cyanogenic glycoside accumulation. *Eucalyptus cladocalyx* allocates up to 20% of leaf nitrogen to accumulation of the cyanogenic glycoside, prunasin (Gleadow et al., 1998). Since cyanogen nitrogen is fully reduced, it does not require additional reduction steps to be assimilated into amino acids. To convert cyanogen nitrogen into amine nitrogen requires the release and rapid assimilation of cyanide from cyanogens. It is assumed that generalized β-glucosidases generate cyanohydrins which then spontaneously decompose to yield cyanide. Cyanide assimilation would then occur via CAS (**Figure 1**) allowing cyanogens to provide reduced nitrogen for protein synthesis (Nartey, 1969; Selmar et al., 1988; Siritunga et al., 2004; Ebbs et al., 2010).

To test this hypothesis, we assessed both the activity of CAS and nitrilase, leading to aspartate and asparagine synthesis, in cassava roots. In addition, we assessed alternate nitrogen assimilation pathways in plants engineered to have very low cyanogen levels. The discovery of genes encoding the cytochrome P450s (*CYP79D1* and *CYP79D2*) that catalyze the first-dedicated step in linamarin synthesis (Andersen et al., 2000; Siritunga and Sayre, 2003; Siritunga et al., 2004) made it possible to design a transgenic approach to reduce cyanogens in cassava. Cassava lines in which linamarin biosynthesis was inhibited in the roots had wild-type linamarin levels in the roots while those in which linamarin biosynthesis was inhibited in the leaves had a 99% reduction of root linamarin levels (Siritunga and Sayre, 2003). This provided confirmation of the leaf as the primary source for root linamarin. We hypothesized that enzymes involved in cyanide assimilation would have preferentially higher activities in the roots compared to the leaves. We detected 3-times higher CAS activity and 1.3-times higher nitrilase activity in cassava roots than in shoots, consistent with cyanide assimilation by CAS. As a corollary to this hypothesis, it would be predicted that competing cyanide assimilation pathways that do not lead to amino acid synthesis would have low activity in cassava roots. It was observed that rhodanese activity was not detected in cassava roots. Thus, there is no apparent competing pathway for cyanide assimilation in cassava roots. There was, however, no significant difference in apparent rhodanese and CAS catalytic turnover activity in cassava leaves suggesting that a substantial portion of CN produced in damaged leaves may be detoxified by rhodanese (Bediako et al., 1981; Ramanujam and Indira, 1984; Siritunga and Sayre, 2003; Siritunga et al., 2004).

If cyanogens are a significant source of reduced nitrogen in plants, a reduction in linamarin synthesis would be expected to impact nitrogen assimilation. Using previously generated low cyanogen plants (Siritunga and Sayre, 2003), we observed elevated nitrate reductase activity in low cyanogen plants relative to wild-type plants suggesting that loss (99%) of cyanogens is compensated by increased nitrate reductase activity in roots. However, elevated root nitrate reductase activity in cassava plants with reduced root linamarin is not sufficient to support plant growth in the absence of supplemental ammonia, further supporting the central role of linamarin turnover in cassava roots as a source of reduced nitrogen for amino acid and protein synthesis (Siritunga and Sayre, 2004).

Over 60% of the reduced nitrogen in stem phloem exudates of cassava is in the form of linamarin (Calatayud and Le Ru, 1996). Thus, reducing the cyanogenic potential of cassava presents a challenge; while toxic to humans, it has an important role in primary nitrogen assimilation. Redirecting cyanogen metabolism toward amino acid and protein synthesis, particularly HNL synthesis in roots to be a nitrogen sink as well as to simultaneously accelerate residual cyanogen (acetone cyanohydrin) turnover during processing, is therefore a more viable option for reducing steady-state linamarin pool sizes, elevating protein content and the nutritional status of cassava roots than blocking linamarin synthesis to reduce cyanide toxicity. Regardless, addressing cyanogen toxicity in cassava roots while supporting active cyanide assimilation into protein remains a complex challenge.

To determine if cyanogen assimilation into proteins could be enhanced by elevating cyanide assimilating enzymes, we overexpressed *CAS* and *NIT4*. Overexpression of *CAS* in cassava roots successfully lead to increased CAS activity, elevated total amino acid pool sizes and increased protein content (+9% relative to wild-type) consistent with elevated CAS activity enhancing cyanide assimilation into amino acids. However, there were unintended consequences of *CAS* overexpression. *CAS* overexpression was associated with poor root development and reduced total fresh weight. At present the mechanism by which plant growth is altered in *CAS* overexpressors is unknown. However, free cyanide has been implicated in plant growth regulation (Smith and Arteca, 2000; Siegien and Bogatek, 2006; Garcia et al., 2010; Xu et al., 2012). Recently, Garcia et al. (2010) have shown that mitochondrial CAS activity is essential for maintaining low cyanide levels essential for root hair development. *CAS Arabidopsis* mutants which accumulate elevated cyanide levels were shown to be defective in root-hair development. This phenotype was rescued by addition of hydroxocobalamin, a cyanide antidote (Garcia et al., 2010). It appears from our studies and studies discussed above that a threshold level of cyanide may be required for proper root development. Reductions in cyanide levels (as expected in transgenic lines with the highest CAS activity) as well as elevated cyanogen levels (*Arabidopsis CAS* mutant) appear to both negatively impact root development. One possible means by which cyanide may impact root development is through regulation of ethylene, and as discussed below IAA production. Previously, Smith and Arteca (2000) have shown that low levels (1 μm) of cyanide enhance transcription of 1-aminocyclopropane-1-carboxylic-acid synthase, the enzyme which mediates the first-dedicated step in ethylene biosynthesis.

Overexpression of *Arabidopsis NIT4* in cassava roots was shown to increase nitrilase activity and alter amino acid pool sizes. However, transgenic plants with the highest NIT activity (>3X wild-type rates) had the lowest total amino acids (50% lower than wild-type), while transgenics having less than a threefold increase in NIT activity had as much as a 50% increase in total amino acids relative to wild-type. These results suggest that there is a complex interplay between NIT enzyme activity and phenotypic response. This is best illustrated by the observed impact of elevated NIT4 activity on cassava growth and development. At least three nitrilase homologs, NIT1, NIT2, and NIT3 are known to be involved in auxin biosynthesis, converting IAN to the plant hormone IAA *in vivo* (Bartling et al., 1992, 1994; Schmidt et al., 1996). *Arabidopsis NIT4*, however, has been reported to not only to have high substrate specificity for cyanoalanine, but also to not recognize IAN as a substrate in the production of IAA (Piotrowski et al., 2001; O’Reilly and Turner, 2003). However, the overexpression of *NIT4* in our studies lead to reduced IAA levels, contrary to expectations, suggesting that cyanoalanine turnover by NIT4 may indirectly impact IAA synthesis. At present the mechanism by which cyanoalanine or cyanide impacts IAA synthesis is not known. However, cyanide is known to stimulate ethylene synthesis which in turn stimulates IAA synthesis and transport (Smith and Arteca, 2000; Normanly, 2007). The complex interplay between ethylene and IAA in regulating plant development with altered cyanide levels may account for impaired root growth in transgenic plants inconsistent with reduction in root IAA levels but consistent with a potential increase in ethylene levels.

## Author Contributions

RS, DS, and TZ were all involved in study design, data acquisition and analysis, as well as manuscript draft and revision.

## Conflict of Interest Statement

The authors declare that the research was conducted in the absence of any commercial or financial relationships that could be construed as a potential conflict of interest.

## Footnotes

1 www.invitrogen.com

2 www.sigmaaldrich.com/

3 www.nitrate.com

4 http://abrc.osu.edu/

5 www.densci.com

6 http://www.graphpad.com/prism/Prism.htm
